# There’s No Sport Without Spectators – Viewing Football Games Without Spectators During the COVID-19 Pandemic

**DOI:** 10.3389/fpsyg.2022.860747

**Published:** 2022-03-31

**Authors:** Ilan Tamir

**Affiliations:** School of Communication, Ariel University, Ariel, Israel

**Keywords:** sport, fans, COVID-19, ghost games, stadiums

## Abstract

The presence of sports fans in the stands is considered a natural and essential element of sporting events. Beyond the atmosphere fans create and the color they add to the game, their presence reflects the idea that the game is more than a competition between two teams —it is a grand battle between communities and identities, which is also the reason that fans are willing to sacrifice so much on behalf of their team. As is other areas of life, the COVID-19 pandemic created an unusual situation, in which sporting events were held without spectators in the stadium stands. In many places around the world, professional sporting activities were permitted but spectators were not allowed into the stadiums due to social distancing restrictions. The current study examines this unique situation and through it, gains an understanding of fans’ beliefs concerning the significance of spectators and their presence in the stadium. Through in-depth interviews with football fans who regularly attend games (in the pre-pandemic period), the current study explores football fans’ experiences as they viewed “ghost games” (where teams played to empty stadiums). Findings show that this unique situation, caused by the global pandemic, heightened fans’ deep-rooted connection to sports and to their favorite team, and also exacerbated the social, emotional, and professional implications of viewing football games with no spectators.

## Theoretical Background

### Sport Fanhood

Sport fanhood is undoubtedly a unique, extremely popular social phenomenon. Millions of fans around the world religiously follow their team week after week (and occasionally at a much higher frequency), on whose behalf they readily make significant sacrifices, including time, money, family interests, and health (Ben-Porat, 2010). For fans, their team is a meaningful and stable element of their lives that accompanies them throughout their lives (Tamir, 2020a).

The reason is that, in contrast to other areas of fanhood, sport fanhood is rooted deeply in fans’ identity. For fans, their team represents much more than a setting for sports events or games. It represents fans’ geographic, cultural, political, ethnic, or familial identity; The team represents the fans themselves and as fans become part of this broader entity, they challenge and contrast their individual identity to the identities of “others.” As sports game often represent a deeper struggle over identity and control, it is also not surprising that sports spectators experience a broad spectrum of emotions (Biscaia et al., 2012). It is on this foundation that we can also understanding the significance that sports fans attribute to competitions that would otherwise be considered as a matter of professional skills alone.

Football stadiums constitute a site of physical encounters for football fans, but they also play a deeper social role in fans’ lives. They constitute platforms that generate a unique atmosphere through which fans’ identity is cultivated and sharpened (Mastromartino et al., 2020), either through chants (Tamir, 2021) or other rituals that help fans understand who they are and, no less importantly, who they are not. In this sense, sports events allow spectators to meet regularly, validating the community to which they belong. It is therefore not surprising that over time, sports stadiums have become a site of political conflicts (Cha, 2009).

Spectators’ presence in the stadium also has a professional impact on what goes on on the field, and the motivation they provide to their teams adds interest and color and even affects referee decisions and the players’ performance on the field (Anderson et al., 2012). Indeed, spectators are often known as the team’s “12th man” (Bilalić et al., 2021).

Fans comprise many different groups with distinct motivations and sense of commitment. Researcher have offered various taxonomies of sports fans (Giulianotti, 2002; van Driel et al., 2019) although it is agreed that true fans are fans who are committed to their team, even during challenging times, and follow their team everywhere. Fans are the club’s “soldiers” (Onwumechili, 2018, p. 113).

### Sports During the COVID-19 Pandemic

COVID-19 is a global pandemic that has affected the world through several waves beginning in late 2019. The virus was initially identified in December 2019 in China and from there it spread rapidly across the world. On March 11, 2020 the World Health Organization (WHO) officially declared a pandemic (Pavli et al., 2020), and in response, many places across the world prohibited social gatherings and events. In some places, full lockdowns were declared, paralyzing social, cultural, and economic systems.

The day the pandemic was declared also became a symbolic date for global sports when the National Basketball Association (NBA) game between the Oklahoma City Thunder and Utah Jazz game was canceled. The NBA season was eventually suspended after a player, Rudy Gobert, tested positive for the virus (Ruihley and Li, 2020). The virus continued to disrupt the world of sports as athletes became infected in increasing numbers and more leagues suspended their activities. The Association of Tennis Professionals (ATP), US Major League Soccer (MLS), Professional Golfers’ Association (PGA), Ladies Professional Golf Association (LPGA), Major League Baseball (MLB), and the National Hockey League (NHL) all postponed their seasons. In a major decision, the top American college sport governing body, the National Collegiate Athletic Association (NCAA), canceled all remaining winter and spring season sporting events, impacting thousands of coaches and athletes. Major international sports events, including UEFA EURO 2020, and the 2020 Olympic and Paralympic Games in Tokyo, were also postponed.

In efforts to maintain some degree of normalcy, many sports organizations across the world decided to hold sports events under specific conditions and restrictions. As a result, many competitions were held, including the Olympic Games (albeit, after a postponement of 1 year) without spectators in the stands, which was an extremely unusual situation in the world of sports.

Despite the relatively short period of time that elapsed since the outbreak of the pandemic, numerous articles have already been written about its effects on the world of sport. Most, quite naturally, address issues that have less to do with the sports themselves, such as finances, the media (Majumdar and Naha, 2020), management, and security (Duckworth et al., 2020).

A study conducted in Austria (Leitner and Richlan, 2021) explored the FC Red Bull Salzburg games that were held without spectators (“ghost games”). This study focused on the games themselves and not the spectators and found that fewer emotional situations arose during games in the absence of spectators, and that, in general, “players and staff acted more factually.” The researchers discovered that ghost games also affected the number of violations and goals in a game. Tamir (2020b) focused on sports spectators in the brief prior in which no sports events were held, and on fans’ sense of deprivation and the effects of a lack of sports events and broadcasts on fans’ lives.

The situation in Israel reflects the state of sports worldwide. The first verified case of COVID-19 was recorded on February 27, 2020 (Itelman et al., 2020), and the government decided on a complete lockdown (the first of three lockdowns) after the virus began to spread. In mid-March, 2020, the Israeli Ministry of Health (MoH) announced that all activities of football and basketball leagues—the country’s two most popular sports–were suspended. The following month, a decision was made to end the season in the lower football leagues. Youth leagues and women’s leagues were similarly suspended.

In May 2020, the MoH published guidelines for the restart of the premier and second football leagues. The league was in fact renewed on May 30, 2020, without spectators, and the games were played at a high degree of frequency (twice weekly) in order to complete the season as quickly as possible. As a result, the decisive stages of Israel’s top league in football took place without spectators in the stadium stands.

The current study focuses on the experience of viewing sports events that have no spectators, through a study of football fans’ experiences as they viewed games without the audience, traditionally considered one of the most important and prominent elements in the game.

## Method

In view of the deep connection between fans and their favorite teams, and their forced separation during the pandemic, at least in terms of the physical manifestations of this relationship, the current study represents an effort to understand the experiences of football fans who viewed their team’s games that took place without spectators in the stands. The forced elimination of this essential element of games and its absence allows us to identify and study its significance.

In-depth semi-structured interviews were conducted with 30 Israeli football fans between the ages of 21 and 52 (mean age 34) in order to gain insights into their experiences during the period of “ghost games.” The gender division of the interviewees (21 male, 9 female) corresponds to the gender division of football fans in Israel (Ben-Porat, 2010). Interviews lasted 30 min on average and were transcribed to allow the researcher to explore interviewees’ responses repeatedly in order to identify patterns. The interviewees, who were used to attend games in person, were asked questions about their feelings at the sight of the empty bleachers such as, Was this sight strange to them? Did the situation trouble them and why? What did they think of when they saw the empty stadium bleachers on TV?

Although researchers have, as noted above, pointed to various categories of sports fanhood (e.g., Giulianotti, 2002; Samra and Wos, 2014), it is conventionally accepted that a “true fan” is a fan who is loyal, is unconditionally connected to the team, is emotionally immersed in the team, and follows it whenever they can. The current study focused on loyal fans of Israeli football teams, who were defined as individuals who had held season tickets for two consecutive years before the outbreak of the COVID-19 pandemic.

An in-depth interview is a central instrument in qualitative research, used by researchers to collect information from interviewees’ perspective, to present multiple interpretations of the phenomenon under investigation (Jones, 2015). Responses are coded into categories and used by the research to identify recurrent patterns and meanings in interviewees’ responses. Analysis of interviewees’ responses allows the researcher to gain a comprehensive understanding of the phenomenon in question, by creating themes that reflect the recurrent explanations for the phenomenon (Turner, 2010).

## Findings and Discussion

### Emotional Effects (“My Body Is Working but My Heart’s Not There”)

Emotion is a dominant element in sports because the competition is the foundation of sports. In sports, sadness and happiness manifest in diverse ways, yet research literature typically addresses emotions in the context of competitions, and the athletes’ emotions, and affords less attention to the spectators’ emotions (Biscaia et al., 2012). Football fans are attributed an important function in the stadium. Beyond the support they give the team, they add color and atmosphere, and provide a tangible dimension to the “competitive struggle” that takes place on the field and constitutes the underlying foundation of all sports competitions. In many places around the world, fans consider themselves part of the team and are consequently known as “the twelfth man.” Fans’ presence is what fuels the rivalry between the teams on the field, and it is not incidental that many of the fans’ chants in a game are expressions of mockery directed at the opponents (Huddleston, 2021). It is therefore not surprising that many fans feel that ghost games that are conducted without fans’ presence feel like encounters stripped of all emotion or suspense.

“I had to do some imagination games, like a kid in kindergarten, to elevate my sense of excitement. It felt similar, but it was so different.”

“…very strange feeling that made it difficult for me to connect to whatever was happening, even to my team. It felt like my body was working but my heart wasn’t there… it felt like a drop in tension and adrenaline.”

The space created by spectators’ absence reduces viewers’ emotional engagement and consequently reduced the significance that fans typically attribute to games.

“Victory is always sweet and a defeat is always painful, but you have to admit that it’s not the same thing without spectators. The reason [for watching the game] is not so strong.”

“To be honest, [our team’s] defeats were less upsetting because I felt that there were no [rival] spectators who danced and cheered our defeat. Suddenly there was much less stress in the games.”

Previous research found that fans reported a “phantom pain” in certain situations (Tamir, 2019). Borrowing from this finding, our findings show that fans experience ghost games as a phantom pain, pain caused by the absence of fans, an essential organ of the team, in the stadium.

“It feels as if the most important thing is simply not there. It really bothered me. How can you watch and enjoy a game like that? It’s not as if we were born into this reality. We know what a real game is like. This situation [the absence of spectators] is intolerable.”

The diminution of fans’ emotional engagement aligns well with the findings of a preliminary study (Leitner and Richlan, 2021), albeit of limited scope, which analyzed games without spectators during the COVID-19 pandemic and found that the players on the field expressed fewer emotional outbursts in the absence of spectators.

### Professional Effects (“It’s Not Really Sports if There Are No Spectators”)

Rivalry is considered one of the main drivers of sports. Games between teams frequently reflect a broader competition over prestige and dominance (Tyler and Cobbs, 2017). The presence of spectators in the stadium constitutes the tangible, physical manifestation of the rivalry between teams. Empty stands largely dismantled the prestige of professional football games. For many viewers, this unusual scene created a tangible sense of discomfort, as if they were watching amateur games. Moreover, this feeling was exacerbated by the fact that several games were relocated to smaller stadiums (larger stadiums were simply not needed with no spectators in the stands). The silence in the stands in the spectators’ absence highlighted the calls exchanged by the players and the coaches. For the viewing fans at home, this merely emphasized the feeling that they were watching a neighborhood game.

“You only hear the coaches’ screams and the screams of the players on the bench. It feels more like watching a football game of eight-graders in the neighborhood park. You know very well what I think about the role of cheering during a game: It’s not really sports if there are no spectators. Period.”

“There’s no sports without an audience…it feels as if they’re not competing over anything. Just like a game with no point.”

“It’s boring and it feels like a practice game that has no importance… the entire prestige of the game is gone. When they started piping in sounds of an audience, it did add something.”

The experiences reported by these fans find support in other findings that show that the absence of spectators affects the professional aspects of a game. A study by Gracenote, a Nielsen subsidiary, found that teams play differently and their playing style is less attractive in games without spectators: There are fewer kicks, fewer dribbles, and fewer goals (Smith, 2020).

One accepted justification for spectators’ presence in sports stadiums is their ability to affect the game, whether this is an objective effect or the fans’ subjective feeling (Moskowitz and Wertheim, 2011). Spectators are strongly motivated to encourage their team, and this apparently sets a unique, professional atmosphere for the game.

### Social Effects

For many fans, games serve as a platform for social encounters as well as platforms for defusing the stress and problems of everyday life. The structure of sports competitions and leagues allows fans to meet on a regular weekly basis, creating communities with unique rituals and a strong sense of commitment (Wann and Jeffrey, 2019). In this respect, sports stadiums are much more than concrete edifices that host events–They represent a way of life and constitute a site that fosters a sense of community (Mastromartino et al., 2020). Fans’ forced absence stripped the stadiums of their social dimension and was the source of significant social deprivation for the fans.

“It feels like I am on the outside, watching the house where my friends and I should be. It feels very strange.”

“Watching the field like that is like looking at an empty candy store. Only shelves, no chocolate. The place makes me homesick but the most important thing is not there. I feel like popping over there, but I suddenly feel alone.”

On the other hand, the sight of empty, abandoned stands, heightened fans’ longing and their desire to return to their familiar social routine. One of the most dominant themes in the fans’ responses is their use of the first-person plural (“we” “us”) when describing their individual responses to the absence of spectators.

“I am looking at the field, and you can’t understand the longing that it generates in me. I image us popping over there and shaking up a storm in the stadium. I really want to go back there.”

“Sometimes I see it and I really try to image what would happen if I were in the stadium in that moment. It’s a feeling that bothers me mainly at the end of a game. You see the team leaving the field and I’m sad that we can’t be there, just pop over there and support [them].”

## Summary

Arsène Wenger, a French former football manager and player who is currently serving as FIFA’s Chief of Global Football Development, stated in a recent interview that it is impossible to imagine an entire season without spectators. Whether intentionally or not, this statement alludes to ghost games’ multiple, deep-seated repercussions for fans. The current study used the unique opportunity, presented by the COVID-19 pandemic, of a natural experiment, in which professional football players compete when fans are not present in the stands. The basic assumption of this study that the significance of a single element can sometimes be more clearly identified and explored in its absence.

The global COVID-19 pandemic has rocked the world of culture, include global sports. After a period of complete suspension of games, leagues in many places around the world were renewed. To prevent the spread of the virus, however, a prohibition was imposed on the presence of fans, who are typically a significant and integral element in the stadium during a game. As a result, for viewers at home, the regular backdrop of football games was replaced by the sight of empty stands.

The current study explored fans’ remote viewing experience of football games that take place without the presence of spectators. The assumption was that the absence of spectators in the stands would provide insights on the significance of fans’ presence in the stadium, from the perspective of the fans themselves. Studies on the effects of the COVID-19 pandemic on sports mainly focused on teams, players, or referees. The current study directs research attention to one of the most important elements in modern sports—the audience.

The findings highlighted the significance of spectators in the stands in three main dimensions: emotional, professional, and social. From an emotional perspective, the absence of spectators dramatically reduced viewers’ emotional engagement, and consequently fans’ pre-game anticipation and post-game euphoria or disappointment. From a professional perspective, fans’ experienced these games as amateur games, to which they felt limited social commitment. The empty stands heightened their longing for the sense of community that characterizes sport fanhood.

## Author Contributions

IT conceived the original idea, developed the theory, contributed to the interpretation and analysis of the results, and to the writing of the manuscript.

## Conflict of Interest

The author declares that the research was conducted in the absence of any commercial or financial relationships that could be construed as a potential conflict of interest.

## Publisher’s Note

All claims expressed in this article are solely those of the authors and do not necessarily represent those of their affiliated organizations, or those of the publisher, the editors and the reviewers. Any product that may be evaluated in this article, or claim that may be made by its manufacturer, is not guaranteed or endorsed by the publisher.
